# Allogeneic mesenchymal stem cells inhibited T follicular helper cell generation in rheumatoid arthritis

**DOI:** 10.1038/srep12777

**Published:** 2015-08-11

**Authors:** Rui Liu, Xia Li, Zhuoya Zhang, Min Zhou, Yue Sun, Dinglei Su, Xuebing Feng, Xiang Gao, Songtao Shi, Wanjun Chen, Lingyun Sun

**Affiliations:** 1 Department of Immunology and Rheumatology, Drum Tower Clinical Medical College of Nanjing Medical University, Nanjing, Jiangsu, 210008, PR China; 2 Department of Immunology, College of Basic Medical Science, Dalian Medical University, Dalian, Liaoning, 116044, PR China; 3 Department of Immunology and Rheumatology, The Affliated Drum Tower Hospital of Nanjing University Medical School, Nanjing, Jiangsu, 210008, PR China; 4 Key Laboratory of Model Animal for Disease Study, Model Animal Research Center, Nanjing University, 12 Xuefu Road, Nanjing 210000, China; 5 Center for Craniofacial Molecular Biology, Ostrow School of Dentistry, University of Southern California, 2250 Alcazar Street, CSA 103, Los Angeles, CA 90033, USA; 6 Mucosal Immunology Section, National Institute of Dental and Craniofacial Research, National Institutes of Health, Bethesda, Maryland, USA

## Abstract

T follicular helper (Tfh) cells provide help for antigen-specific B cells. We have previously shown that Tfh cell frequency was increased and associated with auto-antibodies in patients with rheumatoid arthritis (RA), suggesting a possible involvement of Tfh cells in its pathogenesis. Mesenchymal stem cells (MSCs) represent a promising alternative cell therapy for RA by modulating T and B cell activation and proliferation. However, it remains unknown whether MSCs have immunoregulation on Tfh cells. In this paper, we have demonstrated that allogeneic MSCs could suppress Tfh cell differentiation in RA patients partly via the production of indoleamine 2,3-dioxygenase (IDO). IFNγ generated from Tfh cell differentiation system induced IDO expression on MSCs. MSCs transplantation (MSCT) into collagen-induced arthritis (CIA) mice prevented arthritis progression by inhibiting both the number and function of Tfh cells *in vivo.* These findings reveal a novel suppressive function of MSCs in Tfh cells, which has implication in understanding the underlying mechanisms of the immunotherapeutic effects of MSCs on RA patients.

Recently, T follicular helper (Tfh) cells have emerged as a new T helper lineage specialized in the assistance of B cells during the germinal center (GC) reactions in secondary lymphoid tissues^1^. Tfh cells are characterized by positive expression of chemokine (C-X-C motif) receptor 5 (CXCR5), inducible costimulatory molecule (ICOS), programmed cell death protein (PD)-1, CD40 ligand (CD40L) and the secretion of interleukin (IL)-21, along with decreased expression of CC-chemokine receptor (CCR7)^2^. B cell lymphoma-6 (Bcl-6) is identified as Tfh cell master transcription factor that is necessary and sufficient for the development of Tfh cells *in vivo*^3^,^4^. Tfh cells have recently been shown to support B cells by providing cell survival and proliferative signals to B cells, stimulating their process of somatic hypermutation and helping them differentiation into memory B cells or plasma cells^5^.

Rheumatoid arthritis (RA) is characterized by persistent synovitis and systemic inflammation, frequently leading to cartilage and bone destruction. Although the etiology is still unknown, T and B cells and their interaction with proinflammatory cytokines are considered to participate in the pathophysiology of RA^6^,^7^. Auto-antibodies to citrullinated cyclic peptides (CCP) and rheumatoid factor (RF) have been indicated to be associated with this disease course^8^,^9^,^10^. The production of auto-antibodies by B cells depends on the assistance of Tfh cells, suggesting that auto-reactive B cells might obtain help from Tfh cells in RA patients. We have previously demonstrated that the frequency of circulating Tfh cells was substantially upregulated in RA patients and positively correlated with 28-joint count disease activity score (DAS28) and serum anti-CCP antibody level, suggesting that Tfh cells might be involved in the pathogenesis of RA^11^.

Mesenchymal stem cells (MSCs) are multipotent stromal cells capable of differentiating into different cell lineages including osteoblasts, chondrocytes and adipocytes^12^. In addition to the differentiation potential, their immune suppressive properties by modulating T and B cell proliferation and differentiation as well as dendritic cell maturation have garnered increasing attention^13^,^14^,^15^. Reportedly, MSCs represent a promising cell therapy for autoimmune diseases including experimental and clinical RA^16^,^17^, systemic lupus erythematosus (SLE)^18^,^19^,^20^,^21^, systemic sclerosis (SSc)^22^, and Sjögren’s syndrome (SS)^23^. We have previously shown that refractory RA patients achieved a reduction of erythrocyte sedimentation rate (ESR), DAS28 and pain visual analog scale (VAS) score after allogeneic MSCs transplantation^24^. However, how allogeneic MSCs exert their immunoregulation in RA remains unclear.

## Results

### Umbilical cord (UC)-MSCs suppressed the differentiation and proliferation of Tfh cells

Firstly, to detect whether UC-MSCs have an immunoregulatory role in Tfh cells, we cocultured phytohaemagglutinin (PHA)-stimulated peripheral blood mononuclear cells (PBMCs) with UC-MSCs. The result showed that UC-MSCs suppressed Tfh cell generation in RA and healthy control (HC) PBMCs and this UC-MSCs-mediated suppression was dose-dependent (Supplementary Fig. 1, 2). However, UC-MSCs did not inhibit the generation of CD4^+^CXCR5^−^T cells, and upregulated them both in RA and HC PBMCs (Supplementary Fig. 3), suggesting that CD4^+^CXCR5^+^T cells not CD4^+^CXCR5^−^T cells might be specifically susceptible to UC-MSCs-mediated suppression.

**Figure 1:**
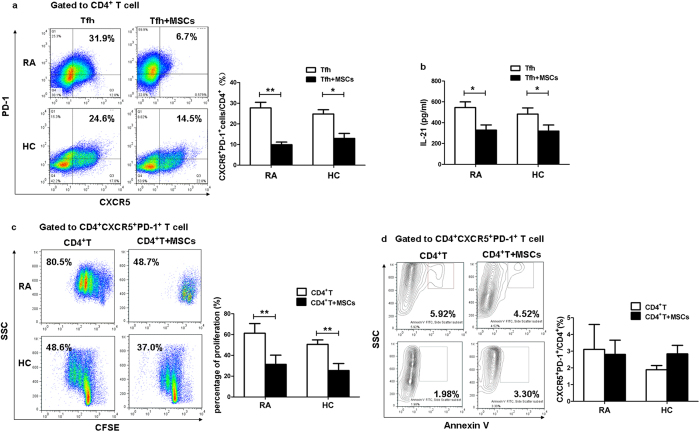
UC-MSCs suppressed the differentiation and proliferation of Tfh cells in RA patients. (**a**) RA and HC naïve CD4^+^T cells (1 × 10^6^/well) were differentiated into Tfh cells under Tfh cell-polarizing condition for 3 days. Then these differentiating Tfh cells were washed with phosphate-buffered saline (PBS) and further expanded alone or cocultured with UC-MSCs (1 × 10^5^/well) in the presence of 3 μg/ml soluble anti-CD3/28 for another 2 days. UC-MSCs inhibited the differentiation of Tfh cells in both RA patients (N = 4) and HC (N = 4). (**b**) The level of IL-21 significantly decreased in the supernatant of each group of Fig. a after 5 days’ coculture (N = 4). (**c**) CD4^+^T cells (1 × 10^6^/well) labeled with CFSE were cocultured with UC-MSCs (1 × 10^5^/well) for 4 days. UC-MSCs inhibited the proliferation of Tfh cells in both RA patients (N = 6) and HC (N = 5). (**d**) CD4^+^T cells (1 × 10^6^/well) were cocultured with UC-MSCs (1 × 10^5^/well) for 3 days. UC-MSC had no effect on the apoptosis of Tfh cells in RA patients (N = 3) or HC (N = 6). **p < 0.01; *p < 0.05.

Next, we sought to identify whether UC-MSCs-mediated immunosuppressive effect could affect Tfh cell differentiation, proliferation or apoptosis. Naïve CD4^+^T cells isolated from RA patients were induced to differentiate into Tfh cells. As Supplementary Fig. 4 showed, enhanced mRNA levels of IL-21 and transcription factor-Bcl-6 accompanied with positive expressions of ICOS, CXCR5 and PD-1 in our differentiation system recognized these induced T cells as circulating Tfh cells. In order to detect the effect of UC-MSCs on Tfh cell differentiation, naïve CD4^+^T cells were stimulated under Tfh cell-polarizing conditions for 3 days and then cocultured with or without UC-MSCs for another 2 days in the presence of anti-CD3/28. We found that the frequency of CD4^+^CXCR5^+^PD-1^+^T cells was substantially reduced, along with lower supernatant IL-21 levels in the presence of UC-MSCs (Fig. 1a,b), suggesting that UC-MSCs inhibited Tfh cell differentiation. Then, CD4^+^T cells were labeled with carboxyfluorescein diacetate succinimidyl ester (CFSE) and cocultured with or without UC-MSCs for 4 days. The result showed that UC-MSCs significantly suppressed Tfh cell proliferation (Fig. 1c). However, no effect of UC-MSCs on Tfh cell apoptosis was observed after 3 days’ coculture (Fig. 1d). To sum up, the data demonstrated that UC-MSCs downregulated Tfh cells through inhibiting their differentiation and proliferation in RA patients.

### The inhibition of Tfh cell differentiation by UC-MSCs may be partly mediated by indoleamine 2,3-dioxygenase (IDO)

**Figure 2:**
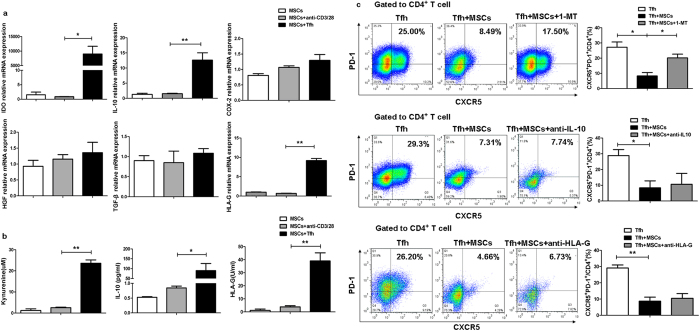
UC-MSCs inhibited Tfh cell differentiation through the release of IDO. (**a**) UC-MSCs were collected after 2 days’ coculture with differentiating RA Tfh cells and then were fixed by Trizol. The mRNA expressions of IDO, IL-10 and HLA-G were upregulated on UC-MSCs when cocultured with RA Tfh cells (N = 4). (**b**) Levels of kynurenine, IL-10 and HLA-G increased in the supernatants of each group of Fig. a (N = 4). (**c**) 100 μM 1-MT or 10 μg/ml anti-IL-10 antibody or 10 μg/ml anti-HLA-G antibody was added to the MSCs-Tfh cells coculture system for 2 days’ culture. 1-MT, but not anti-IL-10 or anti-HLA-G could block the suppressive effect of UC-MSCs on Tfh cells (N = 3). **p < 0.01; *p < 0.05.

Next, we focused on the role of soluble factors in the inhibition of Tfh cell differentiation mediated by UC-MSCs. Strikingly, UC-MSCs expressed extremely higher levels of IDO mRNA when coculturing with differentiating Tfh cell from RA patients (Fig. 2a). IL-10 and human leucocyte antigen-G (HLA-G) mRNA expression were moderately elevated in UC-MSCs (Fig. 2a). Consistent with increased mRNA levels, the IDO enzymatic activity, supernatant IL-10 and HLA-G levels were all significantly enhanced in the supernatant of UC-MSCs-Tfh cells coculture system (Fig. 2b). These results suggested that IDO, IL-10 and HLA-G might be involved in UC-MSCs-mediated suppressive effect on Tfh cells. To confirm that, IDO inhibitor 1-MT or anti-IL-10 antibody or anti-HLA-G antibody was added to the UC-MSCs-Tfh cells cocultures respectively. The results showed that 1-MT partly reversed the immune suppressive effect of UC-MSCs on Tfh cell differentiation. However, the similar effect was not found for anti-IL-10 or anti-HLA-G antibody (Fig. 2c).

### IFNγ promoted the production of IDO by UC-MSCs

**Figure 3:**
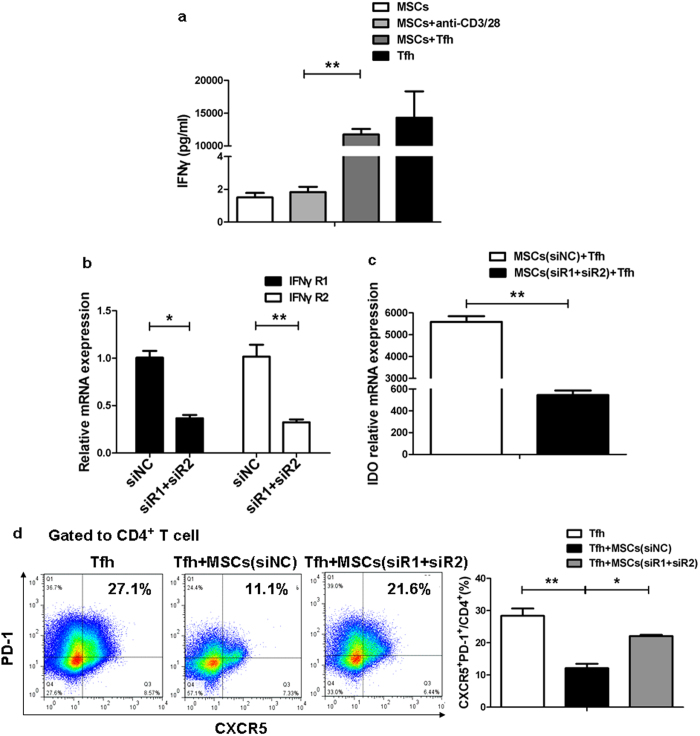
IFNγ promoted IDO production by UC-MSCs. (**a**) The level of IFNγ significantly increased in the supernatant of UC-MSCs and RA differentiating Tfh cells coculture system (N = 5). (**b**) The mRNA expression of IFNγR1 and IFNγR2 downregulated markedly in UC-MSCs transfected with a combination of siR1 and siR2 *versus* UC-MSCs transfected with siNC. (**c**) UC-MSCs (1 × 10^5^/well) with IFNγR1 and IFNγR2 double knockdown were collected after 2 days’ coculture with differentiating Tfh cells and then were fixed by Trizol. These UC-MSCs had lower IDO mRNA expression after cocultured with RA differentiating Tfh cells (N = 3). (**d**) The suspension cells were collected from the coculture system of Fig. c and then analyzed by FACS. UC-MSCs with IFNγR1 and IFNγR2 double knockdown could not suppress the differentiation of Tfh cells effectively in RA patients (N = 3). **p < 0.01; *p < 0.05.

We have previously reported a robust induction of IDO in UC-MSCs by IFNγ produced by activated T cells in SLE^25^. In our experiment, we found that there were great amounts of IFNγ released in UC-MSCs-Tfh cells cocultures (Fig. 3a). To confirm the role of IFNγ in the release of IDO by UC-MSCs, we knocked down the IFNγ receptor (R) with siRNAs targeting IFNγR1 (siR1) and IFNγR2 (siR2) in UC-MSCs. As shown in Fig. 3b, the combination of siR1and siR2 transfection elicited approximately 70% knockdown efficiency compared with control siRNA (siNC). As expected, the downregulation of IFNγR markedly reduced IDO mRNA expression in UC-MSCs and reversed the suppressive effect of UC-MSCs on Tfh cells (Fig. 3c,d). Thus, these results suggested that IFNγ is the key factor to induce IDO production by UC-MSCs.

### UC-MSCs suppressed Tfh cells in mice with collagen induced arthritis (CIA)

**Figure 4:**
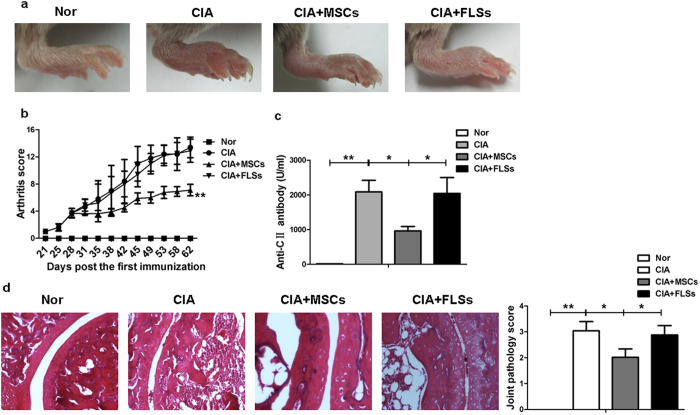
UC-MSCs ameliorated the progression of CIA. (**a**) The severity of CIA was progressively attenuated in UC-MSCs-treated group (N = 5). (**b**) Arthritis score was decreased in UC-MSCs-treated group (N = 5). (**c**) The level of anti-CII antibody was significantly downregulated in UC-MSCs-treated group (N = 5). (**d**) H&E staining exhibited marked improvement of mononuclear cell infiltration, severe synovitis, pannus formation and bone erosion in UC-MSCs-treated group. (N = 5). **p < 0.01; *p < 0.05.

We are interested in investigating the effect of UC-MSCs on Tfh cells *in vivo*. We first showed that adoptive transfer of human UC-MSCs suppressed the progression of CIA, which was demonstrated by reduced swelling on hind limb (Fig. 4a). Importantly, the therapeutic effect was specific to UC-MSCs, as adoptive transfer of the same number of fibroblast-like synoviocytes (FLSs) failed to prevent the progression of arthritis. This was further verified by arthritis scores, the levels of anti-type II collagen (CII) antibody and histologic evaluation (Fig. 4b–d).

**Figure 5:**
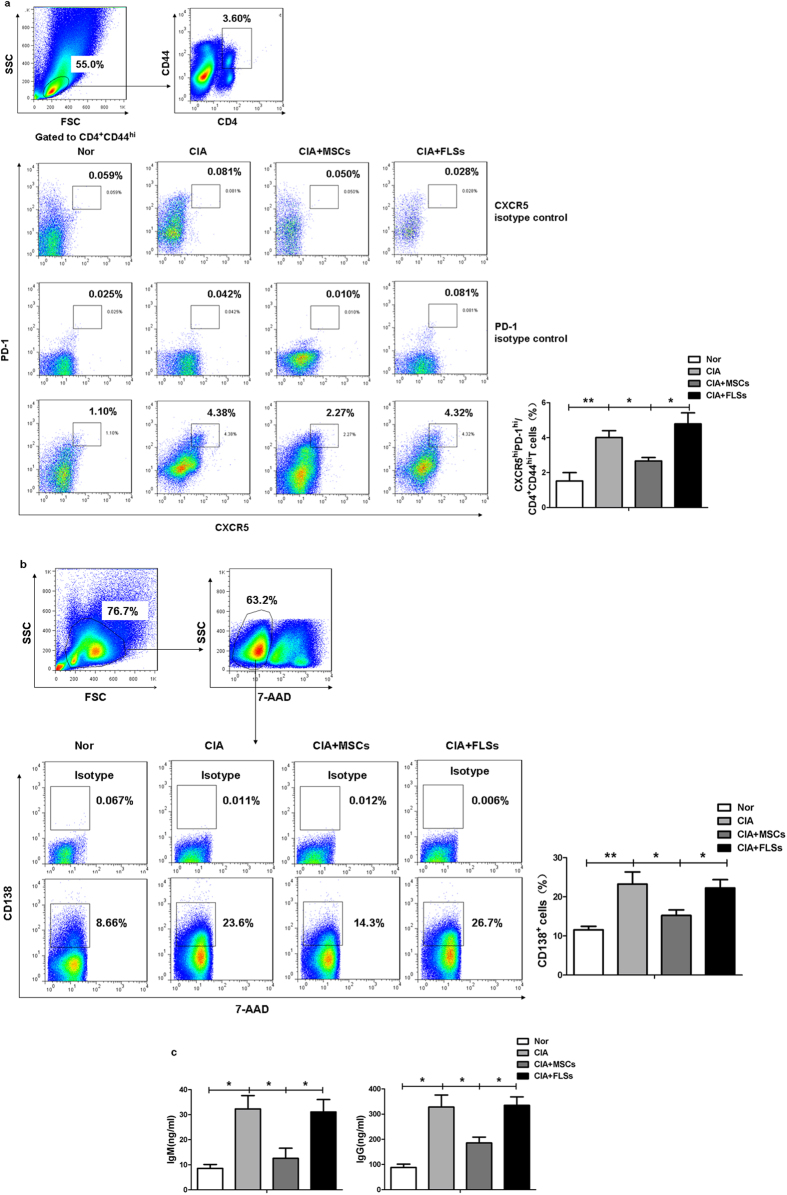
UC-MSCs downregulated both number and function of Tfh cells *in vivo*. (**a**) UC-MSCs infusion decreased the frequency of splenic CD4^+^CD44^hi^CXCR5^hi^PD-1^hi^Tfh cells in CIA mice (N = 5). (**b**) CD4^+^CXCR5^+^T cells (1 × 10^5^ cells/ well) were purified from the spleen of each group and then cocultured with splenic B cells (3 × 10^5^ cells/well) purified from normal mice for 5 days. The frequency of plasma cells were significantly upregulated in UC-MSCs-treated CIA mice after CD4^+^CXC5^+^T cells coculturing with B220^+^B cells (N = 5). (**c**) The levels of supernatant IgG or IgM reduced in the coculture system of CD4^+^CXCR5^+^Tfh **c**ells from each group mice and B cells (N = 5). **p < 0.01; *p < 0.05.

To investigate whether UC-MSCs could inhibit Tfh cells in CIA mice, we examined the frequency of CD4^+^CD44^hi^CXCR5^hi^PD-1^hi^Tfh cells in the spleen. Injection of UC-MSCs significantly downregualted the frequency of Tfh cells (Fig. 5a). In addition, there were lower Th1 and Th17 cell frequencies and higher frequency of CD4^+^CD25^+^Foxp3^+^ regulatory T (Treg) cells in UC-MSCs-treated group (Supplementary Fig. 5).

We then sought to identify whether UC-MSCs could affect the function of Tfh cells *in vivo*. We isolated splenic CD4^+^CXCR5^+^T cells as Tfh cells from each group, and then cocultured them with B220^+^B cells isolated from normal mice. We showed that Tfh cells from UC-MSCs-treated mice had decreased capacity of stimulating B cells to undergo differentiation to plasma cells and the production of IgG and IgM (Fig. 5b,c). The data suggested that UC-MSCs treatment suppressed both the number and function of Tfh cells *in vivo*.

## Discussion

In this study, we showed that allogeneic MSCs inhibited the Tfh cell differentiation and proliferation in RA patients. More importantly, our data revealed that MSCs-mediated inhibition of Tfh cell differentiation was predominantly caused by the production of IDO. In addition, we further showed that both the number and function of Tfh cells were downregulated in CIA mice after MSCT.

Tfh cells are capable of assisting B cells for the production of high-affinity autoantibodies, and the blockade of the ICOS/B7RP-1 pathway, which is high constitutively expressed on Tfh cells or B cells, led to the reduction of Tfh cell and B cell number in GC as well as to the amelioration of disease manifestation in CIA mice^26^. In addition, we have previously shown that the frequency of circulating Tfh cells were markedly increased in RA patients and positively correlated with disease activity and the levels of anti-CCP autoantibody^11^. These findings indicate that Tfh cells might play an important role in the pathogenesis of RA. The seeking for Tfh cell inhibition may be the highlight in RA treatment.

MSCs have attracted extensive attention and been regarded as a novel cell therapy for autoimmune diseases because of their lower immunogenicity and immunomodulatory capacity, especially for T cell regulation. Reportedly, MSCs were capable of inhibiting T cell activation, proliferation and differentiation^27^, as well as balancing Th1/Th2 and Th17/Treg^28^. We have previously shown that refractory RA patients exhibited a remission of symptom by decreasing ESR, DAS28 and VAS score after allogeneic MSCs transplantation^24^. Here, we found that MSCs suppressed the Tfh cell generation of RA patients especially Tfh cell differentiation, and this suppressive effect was partly mediated by IDO, a soluble factor produced by UC-MSCs.

IDO is the rate-limiting enzyme which is involved in the catabolism of the essential amino acid tryptophan into its breakdown product kynurenine^29^. Several animal studies indicated that the deficiency of IDO might contribute to the pathogenesis of RA. For instance, the administration of 1-MT into CIA mice after disease onset exhibited severe paw thickness as well as enhanced humoral and cellular immune responses^30^,^31^. Indo^−/−^ induced arthritis mice showed an earlier onset and increased erosion and cellular infiltration compared to wild-type-induced arthritis. IDO exerts a suppressive effect through the local accumulation of tryptophan metabolites, which blocks T cell growth^32^. In our data, the higher level of IDO existed in Tfh cell differentiation and MSCs cocultures forwarded the assumption that increased IDO expression by MSCs may be a key factor in Tfh cell suppression in RA patients.

Reportedly, IFNγ was able to trigger IDO production by MSCs^25^,^33^ and both activated T cells and Tfh cells could secrete IFNγ^34^,^35^. Intriguingly, IFNγ could also be detected in MSCs-Tfh cells coculture system. Two receptors, IFNγR1 and IFNγR2, both mediated IFNγ effects in target cells^36^. Using specific siRNAs to silence these two receptors in UC-MSCs neutralized the effect of IFNγ on the release of IDO by UC-MSCs, resulting in decreased suppressive ability of UC-MSCs on Tfh cells. Thus, we speculated that IFNγ originated from both activated T cells and Tfh cells in our system induced IDO expression by MSCs.

Although it is still in debate for the therapeutic effects of MSCs on CIA, which might be due to the different experimental systems among the individual laboratories^17^,^37^,^38^,^39^. Our data showed that intravenous injection of allogeneic human UC-MSCs ameliorating CIA mice is in line with the reports of positive therapeutic effects of MSCs on experimental arthritis. The beneficial effect of UC-MSCs on CIA was supported by the reduced number and downregulated function of Tfh cells in the spleen accompanied with decreased Th1 and Th17 cells. Therefore, both *in vitro* and *in vivo* experiments confirm that allogeneic MSCs play an immunoregulatory role in inhibiting Tfh cell number and their function for B cell help in RA microenvironment.

Taken together, our findings showed that UC-MSCs inhibited Tfh cell differentiation through the IDO production in response to IFN-γ in RA patients, which also supposed that RA patients with high IFN-γ levels might be in good response to MSCT. Our study reveals a novel mechanistic insight into how UC-MSCs mediate immune-suppression and will provide supports for the application of UC-MSCs in RA.

## Methods

### Patients and controls

Informed consents followed the declaration of Helsinki and the experimental protocols were approved by Drum Tower Clinical Medical College of Nanjing Medical University. Written informed consent was obtained from all patients. Detailed clinical characteristics were shown in Table 1. All experimental methods applied in this study were carried out according to approved guidelines.

Diagnosis of RA was defined as fulfilling the American College of Rheumatology-European League Against Rheumatism (ACR-EULAR) 2010 criteria for RA at inclusion^40^. Blood samples were collected from RA patients admitted to the ward of Drum Tower Clinical Medical College of Nanjing Medical University. Age and sex matched healthy controls (HC) were obtained from medical examination center.

### Isolation of UC-MSCs and FLSs

Fresh human umbilical cord and synovial tissues were obtained from Drum Tower Clinical Medical College of Nanjing Medical University. UC-MSCs and FLSs were prepared as described previously^21^,^41^.

### Differentiation assay

PBMCs were isolated from peripheral blood using Ficoll density-gradient centrifugation. Naïve CD4^+^T cells were purified from PBMCs according to the manufacturer’s instruction (Miltenyi Biotec, Bergisch Gladbach, Germany). These purified naïve CD4^+^T cells (1 × 10^6^/well) were differentiated into Tfh cells under Tfh cell-polarizing condition (3 μg/ml soluble anti-CD3/28 (eBioscience, San Diego, CA, USA), 50 ng/ml recombinant IL-6 (rIL-6, PeproTech Inc, Rocky Hill, NJ, USA), 50 ng/ml rIL-21 (Abcam, Cambridge, MA, USA), 10 μg/ml anti-IL-4 antibody (eBioscience), 10 μg/ml anti-IFNγ antibody (eBioscience) and 10 μg/ml anti-TGF-β antibody (R&D, Minneapolis, MN, USA)) for 3 days. After initial culture, these differentiating Tfh cells were washed with PBS for 2 times and further expanded alone or cocultured with UC-MSCs (1 × 10^5^/well) in the presence of 3 μg/ml soluble anti-CD3/28 for another 2 days. To detect the factors involved in UC-MSCs-mediated suppression, UC-MSCs were collected after 2 days’ coculture with differentiating Tfh cells and then were fixed by Trizol. Furthermore, 100 μM 1-methyl-DL-tryptophan (1-MT, Sigma), the inhibitor of IDO enzyme activity or 10 μg/ml anti-IL-10 antibody (eBioscience) or 10 μg/ml anti-HLA-G antibody (Biolegend, San Diego, CA, USA) was added to the MSCs-Tfh cells coculture system to block their effects on Tfh cells.

### Proliferation and apoptosis assay

CD4^+^T cells were purified from PBMCs according to the manufacturer’s instruction (Miltenyi). For the proliferation assay, CD4^+^T cells (1 × 10^6^/well) were labeled with 5 μM carboxyfluorescein diacetate succinimidyl ester (CFSE, Invitrogen, Camarillo, CA, USA), and then cocultured with UC-MSCs (1 × 10^5^/well) for 4 days. For the apoptosis assay, CD4^+^T cells (1 × 10^6^/well) were cocultured with UC-MSCs (1 × 10^5^/well) for 3 days and then the cultured CD4^+^T cells were stained with Annexin V (BD PharMingen, San Diego, CA, USA).

### IDO activity assay

Kynurenine metabolites were detected by reverse phase high-performance liquid chromatography (HPLC) as described previously^42^.

### Determination of IL-21, IL-10, HLA-G and IFNγ levels in the supernatant by enzyme-linked immunosorbent assay (ELISA)

IL-21 (4A Biotech Co. Ltd., Beijing, China), IL-10 (Biolegend), HLA-G (Westang Biotech, Shanghai, China) and IFNγ (eBioscience) levels in the supernatant were measured by ELISA kits, according to the manufacturer’s instructions.

### siRNA transfection

siRNA targeting IFNγR1 (siR1; sequence 5’-ACATGTGCTAGTGGATCTA-3’), siRNA targeting IFNγR2 (siR2; sequence 5’-CGAAGATTCGCCTGTACAA-3’) and control nontarget siRNA (siNC) were designed and synthesized by Biomics Biotechnologies (Nantong, Jiangsu, China); UC-MSCs (1 × 10^5^/well) were seeded into 24 well plate in UC-MSCs growth medium supplemented with 10% FBS without antibiotics. After 12 h, siRNA was transfected into UC-MSCs using Lipofectamine® RNAiMAX Reagent (Invitrogen) in Opti-MEM® Medium (Invitrogen) according to the protocol recommended by the manufacturer. Briefly, for IFNγR1 and IFNγR2 double knockdown, 5 pmol of each siRNA (siR1 and siR2) were combined in a final amount of 10 pmol diluted in 50 μl of Opti-MEM® Medium. In a separate tube, 3 ul of Lipofectamine® RNAiMAX Reagent was diluted in 50 μl of Opti-MEM® Medium. The contents of both tubes were mixed by gentle pipetting and then incubated at room temperature for 5 minutes. Then, siRNA-lipid complex was added to UC-MSCs, followed by incubation at 37 °C in a 5% CO_2_ culture incubator for 24 h. Transfected UC-MSCs were either lysed by Trizol or cocultured with differentiating Tfh cells.

### Induction and treatment of CIA

CIA was produced in 6–8 week old male DBA1/J mice (SLRC Laboratory Animal Center, Shanghai, China). Briefly, bovine type II collagen (CII, 4 mg/ml; Sigma) was emulsified with an equal volume of Freund’s complete adjuvant. Mice were injected at the base of the tail with 100 μl of emulsion containing 100 μg of collagen. On day 21, the mice received a booster injection of collagen emulsion in Freund’s incomplete adjuvant. Development of CIA was assessed every 2–3 days by an established macroscopic scoring system^43^. All animal experiments were performed under an institutionally approved protocol for the use of animal research.

The treatment for CIA mice was begun after the onset of disease (Day 28), when CIA model had been established (arthritis score ≥ 1). Mice were injected intravenously with 1 × 10^6^ human UC-MSCs or with 1 × 10^6^ human FLSs. Mice were sacrificed on Day 62.

### Determination of anti-CII antibody and immunoglobulin (Ig)G and IgM by ELISA

The serum levels of anti-CII antibodies (Cayman Chemical), IgG and IgM in the supernatant were measured by ELISA (eBioscience) according to the manufacturer’s instructions.

### Histologic evaluation of CIA

Formalin-fixed limbs were decalcified using standard histologic techniques. Serial 4 μm sections were cut and stained with hematoxylin and eosin and then were analyzed microscopically for the degree of inflammation and bone destruction according to the method reported previously^44^. Each joint was scored separately by two individuals unaware of the treatment protocol.

### Coculture of mice Tfh cells and B cells

Mice CD4^+^T cells were isolated from the splenocytes by negative selection according to the manufacturer’s instructions (Miltenyi). For CD4^+^CXCR5^+^T cell sorting, negative selected CD4^+^T cells were stained with anti-CXCR5 antibody conjugated magnetic beads (Miltenyi), and then they were positive selected. B220^+^B cells were isolated from the splenocytes of DBA1/J mice by positive selection (Miltenyi).

B cells (3 × 10^5^ cells/well) were cocultured with CD4^+^CXCR5^+^T cells (1 × 10^5^ cells/ well) in the presence of 2 μg/ml anti-CD3e, 1 μgCD28 (eBioscience), 2.5 μg/ml CpG 2395 (Invitrogen), 50 ng/ml IL-4 (Peprotech), 5 μg/ml anti-IgM (Jackson ImmunoResearch Lab, West Grove, PA, USA) and 5 μg/ml anti-CD40 (eBioscience) in RPMI 1640 with 10% FBS for 5 days.

### Quantitative RT-PCR

cDNA was synthesized from Trizol-isolated total RNA by use of the SuperScript III First Strand Synthesis SuperMix for quantitative reverse transcribed polymerase chain reaction (qRT-PCR; Takara) according to the manufacturer’s instructions. For real time PCR experiments, reactions containing the SYBR Premix EX Taq^TM^ (Takara), ROX Reference Dye (50 × , Takara), cDNA and gene primers were run on the StepOnePlus^TM^ Real Time PCR Systems and analyzed with StepOne Softwase V2.1 (Applied Biosystems). Relative quantification was calculated using the comparative Ct method^45^. The primers for different genes were listed in Table 2.

### Flow cytometric analysis

Human Tfh cells were stained for surface markers with FITC- or PE-anti-CD4, Alexa Fluor647-anti-CXCR5 and PerCP-Cy5.5-anti-PD-1 (BD). Mice Tfh cells were stained with FITC-anti-CD4, PE-anti-CD44, APC-anti-CXCR5 and PerCP-eFluor®710-anti-PD-1 (eBioscience). Mice plasma cells were stained with APC-anti-CD138 (Miltenyi) and 7-amino-actinomycin D (7-AAD, BD) staining were used to exclude dead cells. Mice Th1, Th2 and Th17 cell staining was described in supplementary files.

### Statistical analysis

Data were summarized as means ± standard error of the mean (SEM). Statistical significance was performed by Student’s t-test. All statistical analyses were performed using GraphPad Prism 5 software (Graph-Pad, San Diego, CA, USA). A *p* value < 0.05 was considered statistically difference.

## Additional Information

**How to cite this article**: Liu, R. *et al.* Allogeneic mesenchymal stem cells inhibited T follicular helper cell generation in rheumatoid arthritis. *Sci. Rep.* **5**, 12777; doi: 10.1038/srep12777 (2015).

## Supplementary Material

Supplementary Information

## Figures and Tables

**Table 1:** Clinical characteristics of 45 RA patients.

Characteristics	Values
Age, yrs	54.7 ± 2.3
Men/women	6/39
Disease duration, yrs	13.1 ± 5.2
DAS28	5.2 ± 0.7
ESR, mm/h	59.4 ± 2.0
CRP, mg/l	34.0 ± 3.8

**Table 2:** The primers for different genes.

Genes	Primers
GAPDH	5′-GCACCGTCAAGGCTGAGAAC-3′(forward)
	5′-TGGTGAAGACGCCAGTGGA-3′(reverse)
Bcl-6	5′-GTTTCCGGCACCTTCAGACT-3′ (forward)
	5′-CTGGCTTTTGTGACGGAAAT-3′ (reverse)
T-bet	5′-AACATCCTGTAGTGGCTGGTG-3′ (forward)
	5′-CCACCTGTTGTGGTCCAAGT-3′ (reverse)
GATA3	5′-TTCCTCCTCCAGAGTGTGGT-3′ (forward)
	5′-AAAATGAACGGACAGAACCG-3′ (reverse)
RORA	5′-TCTCCCTGCGCTCTCCGCAC-3′ (forward)
	5′-TCCACAGATCTTGCATGGA-3′ (reverse)
IL-21	5′-CATGGAGAGGATTGTCATCTGTC-3′ (forward)
	5′-CAGAAATTCAGGGACCAAGTCAT-3′ (reverse)
IDO	5′-GAATGGCACACGCTATGGAA-3′ (forward)
	5′-CAGACTCTATGAGATCAGGCAGATG-3′ (reverse)
HGF	5′-GTCAGCCCTGGAGTTCCATGATA-3′ (forward)
	5′-AGCGTACCTCTGGATTGCTTGTG-3′ (reverse)
TGF-β1	5′-AGCGACTCGCCAGAGTGGTTA-3′ (forward)
	5′-GCAGTGTGTTATCCCTGCTGTCA-3′ (reverse)
IL-10	5′-GAGATGCCTTCAGCAGAGTGAAGA-3′ (forward)
	5′-AGTTCACATGCGCCTTGATGTC-3′ (reverse)
HLA-G	5′-CCTTGCAGCTGTAGTCACTGGA-3′ (forward)
	5′-CACACAGGGCAGCTGTTTCA-3′ (reverse)
COX-2	5′-TGACCAGAGCAGGCAGATGAA-3′ (forward)
	5′-CCACAGCATCGATGTCACCATAG-3′ (reverse)
