# Supplementary material for: Allogeneic mesenchymal stem cells inhibited T follicular helper cell generation in rheumatoid arthritis
Source: Sci Rep. 2015 Aug 11;5:12777. doi: 10.1038/srep12777 (PMC4531289; doi:10.1038/srep12777)
Supplement: Supplementary Information [file srep12777-s1.doc]

Allogeneic mesenchymal stem cells inhibited T follicular helper cell generation in rheumatoid arthritis

Rui Liu^a, 1^, Xia Li^b, 1, 2^, Zhuoya Zhang^c, 1^, Min Zhou^a^, Yue Sun^c^, Dinglei Su^a^, Xuebing Feng^a^, Xiang Gao^d^, Songtao Shi^e^, Wanjun Chen^f^, Lingyun Sun^a, 2^

^a^Department of Immunology and Rheumatology, Drum Tower Clinical Medical College of Nanjing Medical University, Nanjing, Jiangsu, 210008, PR China

^b^Department of Immunology, College of Basic Medical Science, Dalian Medical University, Dalian, Liaoning, 116044, PR China

^c^Department of Immunology and Rheumatology, The Affliated Drum Tower Hospital of Nanjing University Medical School, Nanjing, Jiangsu, 210008, PR China

^d^Key Laboratory of Model Animal for Disease Study, Model Animal Research Center, Nanjing University, 12 Xuefu Road, Nanjing 210000, China

^e^Center for Craniofacial Molecular Biology, Ostrow School of Dentistry, University of Southern California, 2250 Alcazar Street, CSA 103, Los Angeles, CA 90033, USA

^f^Mucosal Immunology Section, National Institute of Dental and Craniofacial Research, National Institutes of Health, Bethesda, Maryland, USA

^1^ These authors contributed equally to this work.

^2^ Correspondence: lingyunsun@nju.edu.cn and lixia416@163.com

Lingyun Sun: Department of Immunology and Rheumatology, Drum Tower Clinical Medical College of Nanjing Medical University, Nanjing, Jiangsu, 210008, PR China

[lingyunsun@nju.edu.cn](mailto:lingyunsun@nju.edu.cn)

Xia Li: Department of Immunology, College of Basic Medical Science, Dalian Medical University, Dalian, Liaoning, 116044, PR China

[lixia416@163.com](mailto:lixia416@163.com)

**Coculture of UC-MSCs with PBMCs**

PBMCs were separated from RA patients or HC. UC-MSCs were cocultured with 2μg/ml phytohemagglutinin (PHA, Sigma)-stimulated PBMCs (1×10^6^) in RPMI 1640 supplemented with 10% FBS.

**Flow cytometric analysis**

For mice Th1, Th2 and Th17 cells, the splenocytes were stimulated *in vitro* with phorbol myristate acetate (PMA, Calbiochem, 50ng/ml), ionomycin (Calbiochem, 500ng/ml) and brefeldin A (Calbiochem, 10μg/ml) for 5 hours, then surface marker of FITC-anti-CD4 and intracellular expression of PE-anti-IL-4, APC-anti-IL-17, PE-anti-IFNγ (eBioscience) were analyzed. Regulatory T cells (Treg) were stained with FITC-anti-CD4 and PE-anti-CD25, and then cells were fixed and permeabilized by Fix/Perm buffer (eBioscience) and stained for anti-forkhead box P3 (Foxp3)-APC (eBioscience).


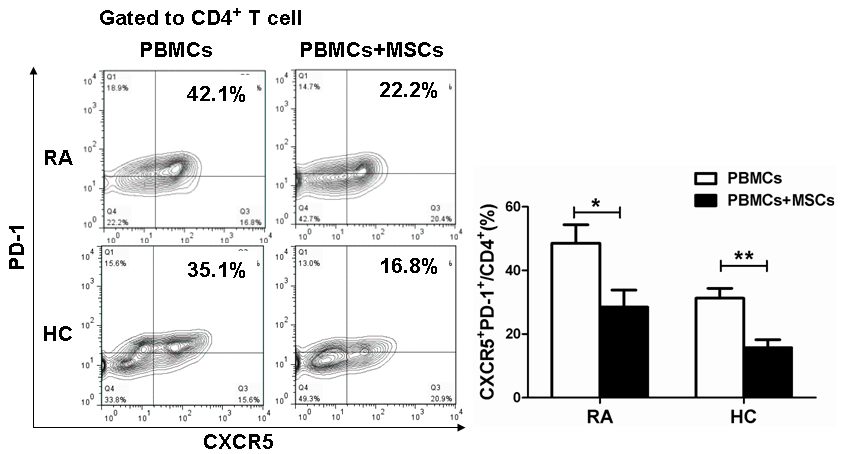


**Figure S1** The percentage of Tfh cells was downregulated when UC-MSCs (1×10^5^/well) were cocultured with activated PBMCs (1×10^6^/well) isolated from both RA patients (N=6) and HC (N=5) for 3 days.


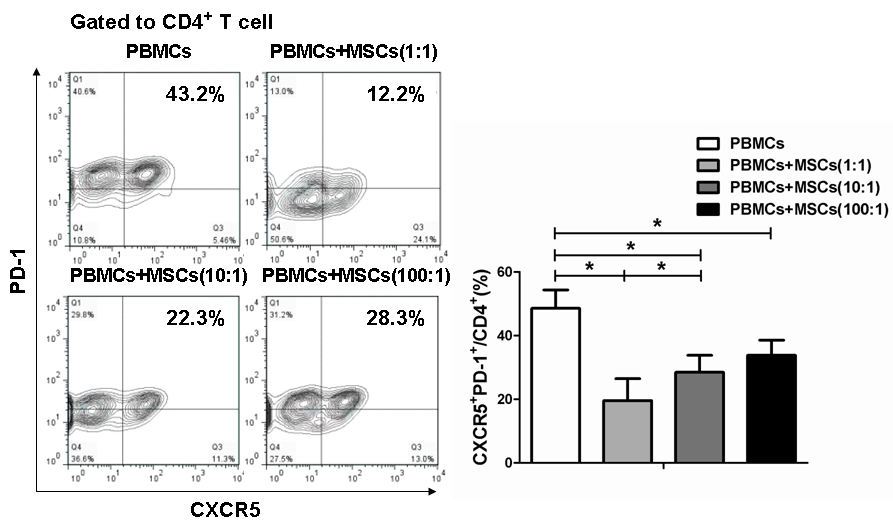


**Figure S2** UC-MSCs dose-dependently suppressed Tfh cell generation in PBMCs of RA patients (N=6) after 3 days’ coculture.


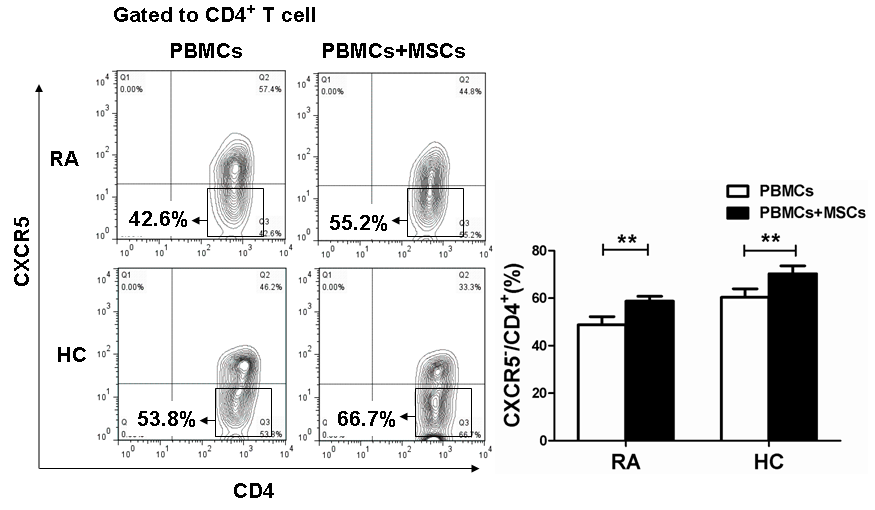


**Figure S3** The percentage of CD4^+^CXCR5^-^ cells was upregulated when UC-MSCs (1×10^5^/well) were cocultured with activated PBMCs (1×10^6^/well) isolated from both RA patients (N=6) and HC (N=5) for 3 days.


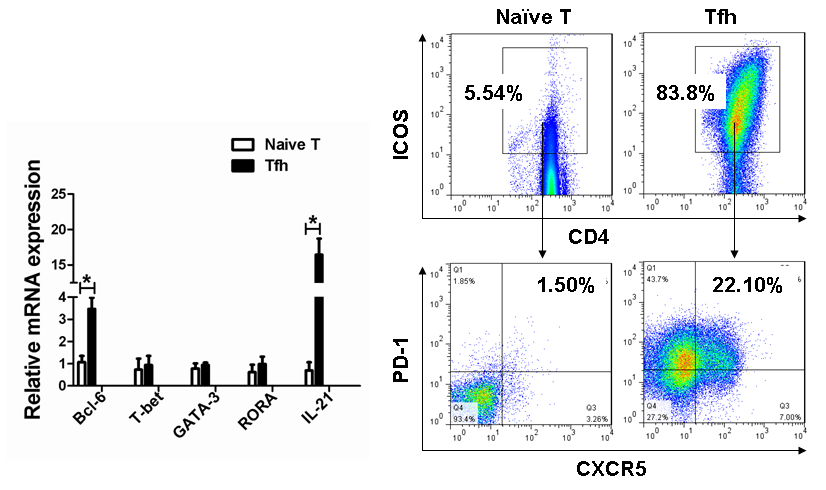


**Figure S4** The mRNA expression of Bcl-6, T-bet, GATA-3, RORA and IL-21 in Tfh cell differentiation system in RA patients after 24 hours’ culture (N=4) and the percentage of CD4^+^CXCR5^+^PD-1^+^ICOS^+^T cells on naïve CD4^+^T cells (1×10^6^/well) under Tfh cell-polarizing condition in RA patients after 5 days’ culture. * *p*<0.05


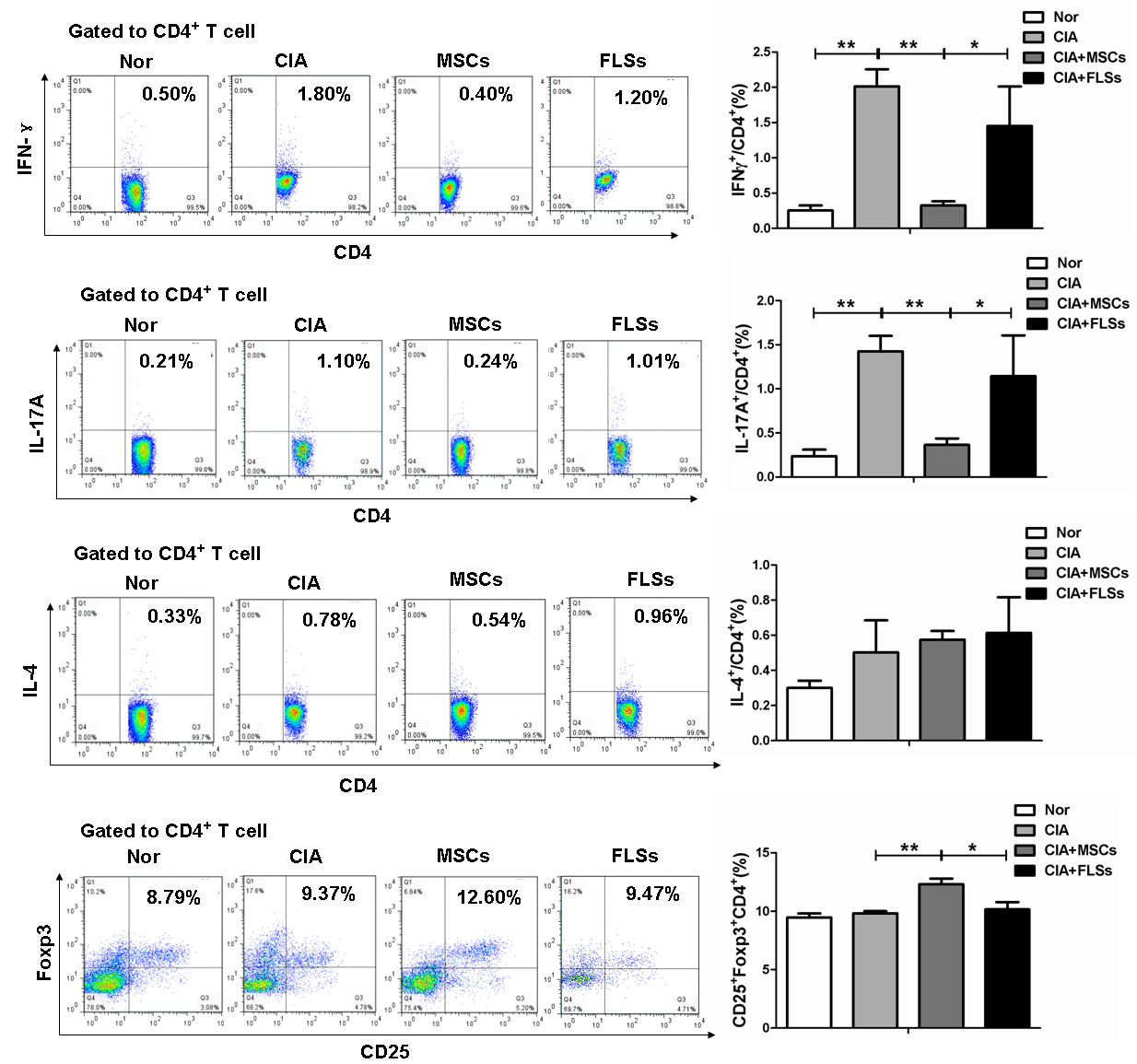


**Figure S5** UC-MSCs infusion decreased the frequencies of Th1, Th2, Th17 cells and increased Treg cells in the splenocytes of CIA mice *in vivo* (N=5).
